# Intestinal perforation during chemotherapeutic treatment of intra-abdominal desmoid tumor in patients with Gardner’s syndrome: report of two cases

**DOI:** 10.1186/s12957-016-0935-0

**Published:** 2016-07-04

**Authors:** Wei Li, Yuhong Zhou, Qian Li, Hanxing Tong, Weiqi Lu

**Affiliations:** Department of Oncology, Zhongshan Hospital, Fudan University, Shanghai, China; Department of General Surgery, Zhongshan Hospital, Fudan University, Shanghai, China

**Keywords:** Desmoid tumors, Gardner’s syndrome, Chemotherapy, Intestinal perforation

## Abstract

**Background:**

A minority of intra-abdominal desmoid tumors is associated with Gardner’s syndrome in which desmoid tumors become an important cause of morbidity and mortality if they are surgically unresectable.

**Case presentation:**

Here, we report two cases of intestinal perforation during chemotherapy in patients with Gardner’s syndrome-associated intra-abdominal desmoids. One female and one male patients who developed inoperable desmoids were given the chemotherapeutic regimen of doxorubicin plus dacarbazine, followed by meloxicam. Significant tumor regression was observed clinically. However, intestinal perforation happened in both patients. They were subjected to emergency surgery, follow-up management, and survived up to now.

**Conclusions:**

The doxorubicin plus dacarbazine chemotherapy is an effective treatment for intra-abdominal demoid tumors in patients with Gardner’s syndrome. On the other hand, given severe adverse events might occur, clinicians should pay more attention that tumor quick regression may cause intestinal perforation in which urgent surgical intervention is necessary.

## Background

Desmoid tumors (aggressive fibromatosis) are rare, locally aggressive, benign fibroblastic tumors, accounting for ~0.03 % of all neoplasms. Although desmoid tumors (DTs) are histologically benign and without any metastatic potential, they are locally aggressive with a tendency to invade nearby structures and recur after resection. The etiology of these tumors is still controversial, but antecedent trauma including surgery, endocrine, and genetic factors seem to be implicated. Most desmoids are sporadic, but some are in association with familial adenomatous polyposis (FAP), also known as Gardner’s syndrome.

Although desmoid tumors can arise at any body site, the desmoids in Gardner’s syndrome usually arise in the abdomen and are a major cause of morbidity and mortality in patients who undergone prophylactic colonic surgery [1, 2]. Desmoids account for 10–14 % of deaths of Gardner’s syndrome patients due to intestinal obstruction or perforation, making it the second leading cause of death after colorectal carcinoma [3, 4]. Complete surgical removal remains the optimal treatment for extra-abdominal and abdominal-wall desmoids but is not recommended for mesenteric desmoids because of the high risk of recurrence and the difficulties involved in the operation [5]. Besides, surgical excision of intra-abdominal desmoids is hazardous, with a perioperative mortality rate of 10 to 60 % and can lead to further tumor progression [6, 7]. Nonoperative therapies include antiestrogen, nonsteroidal anti-inflammatory drugs, chemotherapy, and targeted therapy. Here, we evaluate the efficacy of a combination therapy of doxorubicin (DOX) plus dacarbazine (DTIC), and meloxicam, which was shown effective in patients with Gardner’s syndrome not amenable to surgery [8–10].

## Case presentation

### Case 1

**Fig. 1:**
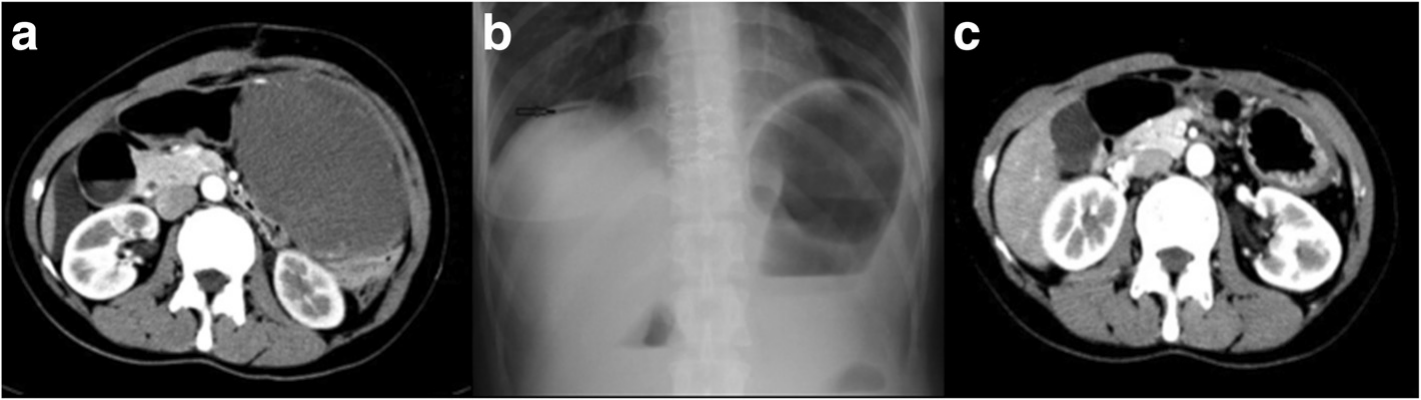
The progression of the intra-abdominal desmoid in patient 1. **a** CT scan before DOX/DTIC therapy showing a large mass in the abdomen in October 2012. **b** X-ray imaging displayed the shadow of free gas (as indicated by the *arrow*) below the right diaphragm. **c** Follow-up CT scan in February 2016

In 2009, a 35-year-old woman (patient 1) was diagnosed as FAP, inherited from her father, with numerous adenomatous polyps at the surface of colon. She did not suffer from osteomas of the skull, thyroid cancer, and epidermoid cysts. The patient received prophylactic colectomy. The patient presented with abdominal bloating and self-palpable left abdominal mass in September 2012 (Fig 1a). Laparotomy showed multiple lumps, with a maximum diameter of 12 cm, which adhered to the abdominal organs. The diagnosis of abdominal mass biopsy indicated desmoid tumors.

Multidisciplinary team (MDT) discussion indicated that the tumors were unresectable. On Oct 11th 2012, the patient was administrated with systemic chemotherapy: doxorubicin (20 mg/m^2^ daily) plus DTIC (150 mg/m^2^ daily) throughout 4 days of drip intravenous infusion every 28 days, followed by meloxicam (10 mg/m^2^). The abdominal bloating was relieved after 1 cycle of chemotherapy. At the end of the second cycle of chemotherapy, the patient felt abdominal pain and fever. X-ray imaging displayed a little shadow of free gas below the right diaphragm (Fig 1b). Blood test showed WBC 22.6 × 10^9^/L, N 89 %. The evidence indicated bowel perforation. Benefit from the tumor shrinkage, intestinal anastomosis and right lower abdominal colostomy were successfully performed. The patient was administrated with meloxicam (7.5 mg bid) after the operation. The lesion remains without tumor progression till now (Fig 1c, more than 40 months so far).

### Case 2

**Fig. 2:**
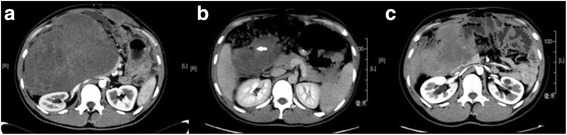
The progression of the intra-abdominal desmoid in patient 2. **a** CT scan before DOX/DTIC therapy showing a large mass in the abdomen and a desmoid tumor on the left abdominal wall. **b** CT scan of the same patient after DOX/DTIC therapy, showing tumor regression and perforation (as indicated by the *white arrow*, the gas was encapsulated by the tumor). **c** Follow-up CT scan in December 2015

A 16-year-old man was found to have multiple polyps by colonoscopy and underwent left half colonic resection in the out court in 2004. He was absent of gastric and duodenal polyps, osteomas, and dermoid cysts. The patient underwent small bowel mesentery root palliative resection, partial ileal resection, and anastomosis plus marginal resection of associated desmoid tumors due to the progression of DT and occurrence of small bowel perforation in February 2014. The DT was enlarged further and led to progressive abdominal pain. The patient presented in our hospital with a great mass (maximum section 18 cm × 13 cm) in abdomen and a desmoid tumor on the left abdominal wall as evidenced by CT scan in April 2014 (Fig 2a). After MDT discussion, the patient was administrated with doxorubicin (20 mg/m^2^ daily) plus DTIC (150 mg/m^2^ daily) throughout 4 days of drip intravenous infusion every 28 days, followed by meloxicam (10 mg/m^2^) on May 24th 2014. After 3 cycles of chemotherapy, abdominal pain symptoms of the patient were disappeared. After 5 cycles of chemotherapy, the patient displayed abdominal pain and high fever with elevated amount of neutrophils. CT scan showed intestinal perforation which was not detected by the X-ray (Fig 2b) and the tumor regression (the maximum section of tumor 14 cm × 8 cm). The patient underwent tumor shrinkage, drainage of focal infection, and intestinal anastomosis. After operation, he was administrated with meloxicam (7.5 mg bid). The treatment is ongoing, and the lesion remains without tumor progression up to now (more than 26 months so far).

### Discussion

About 10 % of Gardner’s syndrome patients develop mesenteric desmoids [11]. In contrast to sporadic DTs, the majority of tumors in patients with Gardner’s syndrome present intra-abdominally. Surgery is the widely accepted first-line treatment for extra-abdominal and abdominal-wall desmoids but is not recommended for intra-abdominal desmoids given the operational difficulties and high risk of recurrence [12, 13]. American Society of Colon and Rectal Surgeons therefore advocate conservative management over initial resection for patients with Gardner’s syndrome or with large slowly growing desmoids compromising the mesentery, vessels, or adjacent organs [14, 15]. Both our patients developed desmoid tumors after prophylactic colectomy of Gardner’s syndrome and presented with abdominal symptoms caused by the compression of the huge mass, indicating a demand for clinical intervention.

Choosing optimal therapy for patients with Gardner’s syndrome is difficult because the diagnosis is rare and no randomized and prospective trials for different treatment approaches are available. In addition, the evaluation of efficacy is problematic given that desmoids have a variable natural history, with some tumors showing spontaneous regression in the absence of treatment. Therefore, the treatment plans have to consider that the growth of DT can be highly variable with growing, stabilization, and even regression. Nonsteroidal anti-inflammatory drugs (NSAIDs) and antiestrogens may be used as first-line therapies for an unresectable tumor [16]. However, NSAIDs and hormone therapy have shown limited success in intra-abdominal desmoids [17, 18]. Cytotoxic chemotherapy is recently investigated for treating such cases. It has been reported that cytotoxic-chemotherapy benefits about 20 to 75 % of treated desmoid patients [19]. Several studies indicated the efficacy and safety of the chemotherapy regimen of doxorubicin and dacarbazine combination in treating patients with Gardner’s syndrome-associated desmoid tumors [8, 9, 20, 21]. Gega combined DOX, DTIC, and meloxicam in the treatment of Gardner’s syndrome-associated desmoids and achieved a great success with high rates of partial or complete response [8]. Adapted the regimen applied by Gega in our study, we observed the remission of symptom in patient 1 and significant tumor shrinkage (partial response) in patient 2, which supports the efficacy of this modality. Unexpectedly, bowel perforation happened in both patients during the treatment. Postoperative pathology analyses showed necrosis of the desmoids. To our knowledge, this is the first time to report such a drastic adverse event resulting from DOX-DTIC-meloxicam in the treatment of Gardner’s syndrome-associated desmoids. It is likely due to the sensitivity to chemotherapy and acute necrosis of the desmoids as indicated by significant tumor regression. Fortunately, we were able to perform intestinal anastomosis in time by the support of MDT. Patients recovered well from the surgery and won the opportunity for further therapy. Inhibited by chemotherapy, the fast growing tumor became amenable to palliative surgery and the residual tumor could be controlled by NASIDs. Alternatively, we speculate that reduction of the dosage may eliminate the adverse effect of DOX-DTIC-meloxicam. In fact, Yamaoto employed low-dose DOX-DTIC therapy to three patients against intra-abdominal desmoids, which was a safe and effective regimen and permitted repeated administration cycles (50 mg/m^2^ DOX and 600–700 mg/m^2^ DTIC per cycle) up to 10–11 times [21].

## Conclusions

In conclusion, we reported two cases of unresectable intra-abdominal desmoid tumors associated with Gardner’s syndrome. Our study provides further evidence of the remarkable efficacy of the DOX-DTIC regimen. However, the drastic regression of tumor upon DOX-DTIC treatment resulted in the perforation of implicated intestine. Even if there is no evidence of intestinal perforation in X-ray imaging during the management of intra-abdominal desmoids complicating Gardner’s syndrome, oncologists should be aware of the possibility of perforation when the patients have high fever and abdominal pain. MDT discussion and team work may be helpful. The modality of DOX-DTIC-meloxicam requires further study on a clinical trial basis. We are also trying a sequential chemotherapeutic regimen of DTIC followed by doxorubicin plus NASID. New results are expected in the near future.

## Abbreviations

DOX, doxorubicin; DTIC, dacarbazine; DTs, desmoid tumors; FAP, familial adenomatous polyposis; MDT, multidisciplinary team; NSAIDs, nonsteroidal anti-inflammatory drugs
